# *catena*-Poly[bis­(dibenzyl­ammonium) [[dichloridomercurate(II)]-μ-sulfato-κ^2^ *O*:*O*′]]

**DOI:** 10.1107/S1600536812006927

**Published:** 2012-02-29

**Authors:** Mouhamadou Sembene Boye, Aminata Diasse-Sarr, Arnaud Grosjean, Philippe Guionneau

**Affiliations:** a Département de Chimie, Faculté des Sciences et Techniques, Université Cheikh Anta Diop, Dakar, Senegal; b CNRS, Université de Bordeaux, ICMCB, 87 Avenue du Docteur A. Schweitzer, F-33608 Pessac, France

## Abstract

The structure of the title compound, (C_14_H_16_N)_2_[HgCl_2_(SO_4_)], consists of an infinite chain propagating along the *c* direction, containing Hg^II^ ions tetra­coordinated by two bridging O atoms of bis-monodentate sulfate anions and two chloride ligands. In the the crystal, N—H⋯O hydrogen bonding between the cations and the anionic chains consolidates the packing. The crystal structure was determined from an inversion twin with approximately equal twin domains.

## Related literature

For the behavior of sulfate as a ligand, see: Sall *et al.* (1992 ▶); Diop *et al.* (2000 ▶); Boye *et al.* (2007 ▶). For the IR vibrational frequencies of sulfate, see: Nakamoto (1978 ▶).
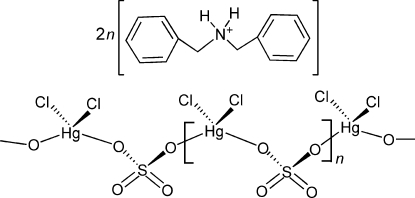


## Experimental

### 

#### Crystal data

(C_14_H_16_N)_2_[HgCl_2_(SO_4_)]
*M* *_r_* = 764.14
Monoclinic, 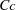

*a* = 22.8275 (5) Å
*b* = 12.9547 (3) Å
*c* = 10.1512 (3) Å
β = 92.095 (2)°
*V* = 2999.94 (13) Å^3^
*Z* = 4
Mo *K*α radiation
μ = 5.41 mm^−1^
*T* = 293 K
0.40 × 0.25 × 0.25 mm

#### Data collection

Nonius Kappa CCD diffractometer
Absorption correction: empirical (using intensity measurements) (*SCALEPACK*; Otwinowski & Minor, 1997 ▶) *T*_min_ = 0.221, *T*_max_ = 0.345
9151 measured reflections
5464 independent reflections
5304 reflections with *I* > 2σ(*I*)
*R*_int_ = 0.020

#### Refinement

*R*[*F* ^2^ > 2σ(*F* ^2^)] = 0.046
*wR*(*F* ^2^) = 0.121
*S* = 1.02
5464 reflections
315 parameters
2 restraints
H-atom parameters constrained
Δρ_max_ = 1.12 e Å^−3^
Δρ_min_ = −2.40 e Å^−3^

### 

Data collection: *COLLECT* (Nonius, 2003 ▶); cell refinement: *SCALEPACK* (Otwinowski & Minor, 1997 ▶); data reduction: *DENZO* (Otwinowski & Minor, 1997 ▶); program(s) used to solve structure: *SHELXS86* (Sheldrick, 2008 ▶); program(s) used to refine structure: *SHELXL97* (Sheldrick, 2008 ▶); molecular graphics: *ORTEP-3* (Farrugia, 1997 ▶); software used to prepare material for publication: *publCIF* (Westrip, 2010 ▶).

## Supplementary Material

Crystal structure: contains datablock(s) I, global. DOI: 10.1107/S1600536812006927/fi2124sup1.cif

Structure factors: contains datablock(s) I. DOI: 10.1107/S1600536812006927/fi2124Isup2.hkl

Additional supplementary materials:  crystallographic information; 3D view; checkCIF report

## Figures and Tables

**Table 1:** Hydrogen-bond geometry (Å, °)

*D*—H⋯*A*	*D*—H	H⋯*A*	*D*⋯*A*	*D*—H⋯*A*
N1—H1*A*⋯O1^i^	0.90	2.44	2.920 (9)	114
N1—H1*A*⋯O3^i^	0.90	2.29	3.037 (10)	141
N1—H1*B*⋯O3^ii^	0.90	1.90	2.766 (9)	161
N2—H2*C*⋯O4^iii^	0.90	2.32	3.043 (10)	137
N2—H2*C*⋯O1^iii^	0.90	2.12	2.857 (9)	139

Symmetry codes: (i) 
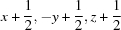
; (ii) 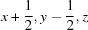; (iii) 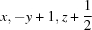.

**Fig. 1.**
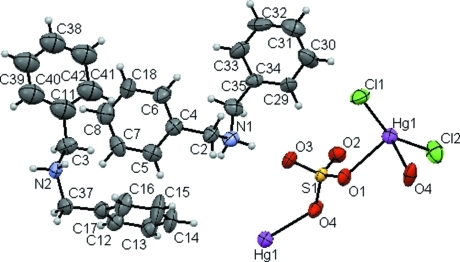
Molecular structure of the title compound. Displacement ellipsoids are drawn at the 50% probability level.

**Fig. 2.**
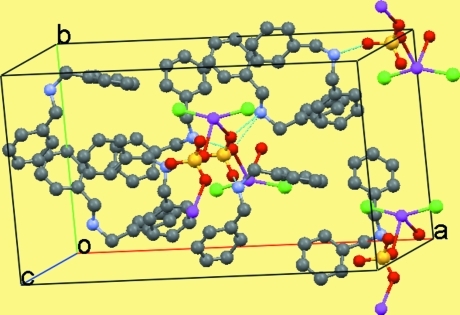
Molecular packing viewed along the c axis. H atoms have been omitted for clarity.

**Fig. 3.**
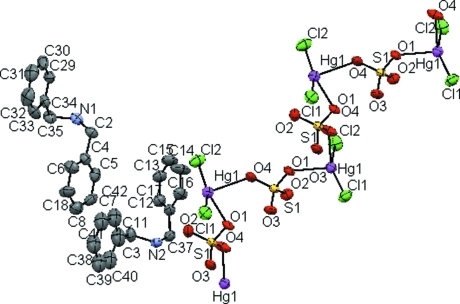
Chains propagate along the c direction. H atoms have been omitted for clarity.

## supplementary crystallographic information

### Comment

In the framework of our research work for understanding the behavior of sulfate acting as ligand (Sall *et al.*, 1992; Diop *et al.*, 2000; Boye *et al.*, 2007), we report the crystallographic study of 2[(C_6_H_5_CH_2_)_2_NH_2_]^+^[HgSO_4_Cl_2_]^2-^.

The structure obtained by single-crystal XRD (Fig. 1) indicate an infinite chain in which each Hg atom is tetracoordinated by two O atoms of two sulfates and two chloride atoms in a distorted tetrahedral geometry. The tetrahedral angles are in the range 80.8 (2)–152.42 (10). The sulfate behaves as a bidentate anion with disparate Hg—O distances [Hg—O(1) = 2.433 (6) and Hg—O(4) = 2.533 (7) Å]. The S—O distances vary from 1.446 (6) to 1.492 (7) Å, the S—O distances for two O atoms linked to Hg atoms [1.474 (7)–1.492 (5) Å] are longer than those non-bonding [1.446 (6)–1.465 (6) Å].

The behavior of bidentate sulfate (C_2v_ symmetry) is confirmed by the infrared data, ν_s_(SO_4_^2-^) appears at 988 cm^-1^ (Nakamoto, 1978) and ν_as_(SO_4_^2-^) splits into three bands (1115, 1082, 1041 cm^-1^). The crystal packing of the title compound is shown in Fig.2.

### Experimental

(C_6_H_5_CH_2_)_2_NH, H_2_SO_4_ and HgCl_2_ (Aldrich chemicals) were used without further purification.

The title compound was obtained by mixing ethanolic solutions of (C_6_H_5_CH_2_)_2_NH (17.66 mmol), H_2_SO_4_ (8.83 mmol) and HgCl_2_ (4.41 mmol) in a 8–4–1 ratio. The mixture was stirred for around two hours at room temperature. Suitable crystals for X-ray diffraction were obtained after slow solvent evaporation. (m.p. 459 K).

The title compound was isolated according to the following reaction:

2(C_6_H_5_CH_2_)_2_NH + H_2_SO_4_ + HgCl_2_→ 2[(C_6_H_5_CH_2_)_2_NH_2_]^+^ [HgSO_4_Cl_2_]^2-^

- Infrared data (cm^-1^) [*vs* = very strong; s = strong]

988 s ν_s_(SO_4_^2-^); 1115 s, 1082 s, 1041 s ν_as_(SO_4_^2-^); 454 s δ_s_(SO_4_^2-^); 610 vs δ_as_(SO_4_^2-^).

### Refinement

Inversion twin matrix instruction was used during refinement. The twin components were 0.498 (9) and 0.502 (9), respectively.

All H atoms were placed in geometrically calculated positions (C—H = 0.93 Å for phenyl H and 0.97 Å for methyelene H, N—H = 0.90 Å) and refined using a riding model with U_iso_(H) = 1.2U_eq_ of the respective carrier atom.

### Crystal data

**Table:**

(C_14_H_16_N)_2_[HgCl_2_(SO_4_)]	*F*(000) = 1504
*M**_r_* = 764.14	*D*_x_ = 1.692 Mg m^−^^3^
Monoclinic, *C**c*	Mo *K*α radiation, λ = 0.71073 Å
Hall symbol: C-2yc	Cell parameters from 10249 reflections
*a* = 22.8275 (5) Å	θ = 0.4–26.0°
*b* = 12.9547 (3) Å	µ = 5.41 mm^−^^1^
*c* = 10.1512 (3) Å	*T* = 293 K
β = 92.095 (2)°	Prism, colourless
*V* = 2999.94 (13) Å^3^	0.40 × 0.25 × 0.25 mm
*Z* = 4	

### Data collection

**Table:**

Nonius Kappa CCD diffractometer	5464 independent reflections
Radiation source: fine-focus sealed tube	5304 reflections with *I* > 2σ(*I*)
Graphite monochromator	*R*_int_ = 0.020
φ and ω scans	θ_max_ = 26.0°, θ_min_ = 2.7°
Absorption correction: empirical (using intensity measurements) (*SCALEPACK*; Otwinowski & Minor, 1997)	*h* = −27→28
*T*_min_ = 0.221, *T*_max_ = 0.345	*k* = −15→15
9151 measured reflections	*l* = −12→12

### Refinement

**Table:**

Refinement on *F*^2^	Secondary atom site location: difference Fourier map
Least-squares matrix: full	Hydrogen site location: inferred from neighbouring sites
*R*[*F*^2^ > 2σ(*F*^2^)] = 0.046	H-atom parameters constrained
*wR*(*F*^2^) = 0.121	*w* = 1/[σ^2^(*F*_o_^2^) + (0.099*P*)^2^ + 6.3836*P*] where *P* = (*F*_o_^2^ + 2*F*_c_^2^)/3
*S* = 1.02	(Δ/σ)_max_ = 0.005
5464 reflections	Δρ_max_ = 1.12 e Å^−^^3^
315 parameters	Δρ_min_ = −2.40 e Å^−^^3^
2 restraints	Extinction correction: *SHELXL97* (Sheldrick, 2008), Fc^*^=kFc[1+0.001xFc^2^λ^3^/sin(2θ)]^-1/4^
Primary atom site location: structure-invariant direct methods	Extinction coefficient: 0.0063 (3)

### Special details

**Table:**

Geometry. All s.u.'s (except the s.u. in the dihedral angle between two l.s. planes) are estimated using the full covariance matrix. The cell s.u.'s are taken into account individually in the estimation of s.u.'s in distances, angles and torsion angles; correlations between s.u.'s in cell parameters are only used when they are defined by crystal symmetry. An approximate (isotropic) treatment of cell s.u.'s is used for estimating s.u.'s involving l.s. planes.
Refinement. Refinement of *F*^2^ against ALL reflections. The weighted *R*-factor *wR* and goodness of fit *S* are based on *F*^2^, conventional *R*-factors *R* are based on *F*, with *F* set to zero for negative *F*^2^. The threshold expression of *F*^2^ > 2σ(*F*^2^) is used only for calculating *R*-factors(gt) *etc*. and is not relevant to the choice of reflections for refinement. *R*-factors based on *F*^2^ are statistically about twice as large as those based on *F*, and *R*-factors based on ALL data will be even larger.

### Fractional atomic coordinates and isotropic or equivalent isotropic displacement parameters (Å^2^)

**Table:**

	*x*	*y*	*z*	*U*_iso_*/*U*_eq_	
S1	0.47859 (7)	0.47550 (14)	0.25406 (15)	0.0294 (3)	
O1	0.4797 (2)	0.4792 (5)	0.1074 (4)	0.0377 (11)	
O3	0.4174 (2)	0.4818 (5)	0.2924 (6)	0.0427 (12)	
O2	0.5059 (3)	0.3804 (5)	0.2988 (6)	0.0460 (13)	
N1	0.8594 (3)	−0.0322 (6)	0.5261 (7)	0.0454 (15)	
H1A	0.8729	0.0132	0.5870	0.054*	
H1B	0.8851	−0.0336	0.4610	0.054*	
C2	0.8012 (4)	0.0059 (8)	0.4703 (8)	0.0452 (18)	
H2A	0.7860	−0.0443	0.4070	0.054*	
H2B	0.8076	0.0698	0.4231	0.054*	
C4	0.7555 (4)	0.0246 (7)	0.5720 (8)	0.0428 (17)	
C6	0.7115 (5)	−0.0450 (9)	0.5881 (11)	0.057 (2)	
H6	0.7110	−0.1064	0.5406	0.068*	
C5	0.7568 (5)	0.1163 (8)	0.6417 (9)	0.051 (2)	
H5	0.7867	0.1639	0.6307	0.061*	
C7	0.7123 (5)	0.1359 (8)	0.7291 (11)	0.057 (2)	
H7	0.7122	0.1980	0.7750	0.068*	
C8	0.6683 (5)	0.0643 (9)	0.7486 (12)	0.063 (3)	
H8	0.6400	0.0764	0.8102	0.075*	
C11	0.5329 (10)	0.1734 (10)	0.580 (2)	0.0868 (16)	
C17	0.6597 (4)	0.4041 (7)	0.4804 (10)	0.0476 (19)	
C16	0.6563 (4)	0.3853 (10)	0.3472 (10)	0.059 (3)	
H16	0.6204	0.3923	0.3015	0.071*	
C13	0.7640 (4)	0.3654 (9)	0.4791 (12)	0.055 (2)	
H13	0.8000	0.3616	0.5244	0.067*	
C14	0.7590 (5)	0.3438 (12)	0.3478 (13)	0.067 (3)	
H14	0.7915	0.3210	0.3035	0.081*	
C15	0.7054 (5)	0.3559 (16)	0.2797 (13)	0.086 (4)	
H15	0.7024	0.3443	0.1892	0.103*	
C12	0.7138 (4)	0.3934 (8)	0.5433 (10)	0.050 (2)	
H12	0.7168	0.4055	0.6336	0.060*	
C18	0.6675 (5)	−0.0243 (10)	0.6755 (13)	0.067 (3)	
H18	0.6371	−0.0713	0.6843	0.081*	
C29	0.8848 (4)	−0.2461 (7)	0.3960 (9)	0.0486 (19)	
H29	0.9186	−0.2067	0.3883	0.058*	
C30	0.8736 (8)	−0.3307 (8)	0.3096 (13)	0.069 (4)	
H30	0.9000	−0.3463	0.2449	0.083*	
C31	0.8241 (7)	−0.3898 (10)	0.3207 (14)	0.074 (3)	
H31	0.8170	−0.4461	0.2656	0.088*	
C33	0.7948 (5)	−0.2806 (11)	0.4996 (12)	0.064 (3)	
H33	0.7671	−0.2645	0.5613	0.076*	
C32	0.7861 (6)	−0.3641 (9)	0.4130 (17)	0.078 (4)	
H32	0.7524	−0.4037	0.4201	0.094*	
C34	0.8456 (4)	−0.2227 (7)	0.4910 (7)	0.0407 (16)	
C35	0.8576 (5)	−0.1388 (8)	0.5890 (9)	0.051 (2)	
H35A	0.8949	−0.1522	0.6348	0.061*	
H35B	0.8274	−0.1397	0.6538	0.061*	
N2	0.5594 (3)	0.3587 (6)	0.5478 (7)	0.0403 (14)	
H2C	0.5291	0.3831	0.5927	0.048*	
H2D	0.5470	0.3490	0.4636	0.048*	
C37	0.6092 (4)	0.4390 (7)	0.5523 (10)	0.0467 (19)	
H37A	0.5948	0.5033	0.5144	0.056*	
H37B	0.6212	0.4521	0.6434	0.056*	
C40	0.4857 (9)	0.1670 (12)	0.6625 (18)	0.0868 (16)	
H40	0.4829	0.2140	0.7312	0.104*	
C42	0.5402 (8)	0.1009 (11)	0.4852 (15)	0.0868 (16)	
H42	0.5723	0.1030	0.4312	0.104*	
C38	0.4515 (8)	0.0193 (11)	0.5571 (16)	0.0868 (16)	
H38	0.4247	−0.0347	0.5558	0.104*	
C39	0.4449 (8)	0.0952 (12)	0.6454 (15)	0.0868 (16)	
H39	0.4114	0.0973	0.6948	0.104*	
C41	0.4946 (8)	0.0166 (11)	0.4705 (14)	0.0868 (16)	
H41	0.4963	−0.0340	0.4057	0.104*	
O4	0.5118 (3)	0.5651 (6)	0.3063 (7)	0.0540 (17)	
C3	0.5785 (5)	0.2590 (8)	0.6051 (10)	0.052 (2)	
H3A	0.5854	0.2670	0.6993	0.063*	
H3B	0.6152	0.2387	0.5673	0.063*	
Cl1	0.40170 (12)	0.2706 (3)	−0.0297 (3)	0.0596 (7)	
Cl2	0.59979 (13)	0.2769 (3)	0.0059 (4)	0.0672 (8)	
Hg1	0.50000 (3)	0.315360 (17)	0.00102 (5)	0.04119 (15)	

### Atomic displacement parameters (Å^2^)

**Table:**

	*U*^11^	*U*^22^	*U*^33^	*U*^12^	*U*^13^	*U*^23^
S1	0.0266 (7)	0.0391 (8)	0.0226 (7)	−0.0018 (6)	0.0036 (6)	0.0009 (6)
O1	0.041 (3)	0.054 (3)	0.018 (2)	−0.003 (2)	0.0051 (19)	−0.002 (2)
O3	0.033 (3)	0.055 (3)	0.041 (3)	−0.003 (2)	0.011 (2)	0.005 (2)
O2	0.048 (3)	0.042 (3)	0.048 (3)	0.001 (2)	−0.001 (2)	0.009 (2)
N1	0.045 (4)	0.054 (4)	0.036 (3)	−0.011 (3)	0.000 (3)	−0.004 (3)
C2	0.040 (4)	0.059 (5)	0.036 (4)	−0.006 (4)	0.001 (3)	−0.001 (4)
C4	0.044 (4)	0.052 (5)	0.032 (3)	−0.001 (3)	−0.006 (3)	0.005 (3)
C6	0.048 (5)	0.066 (6)	0.057 (5)	−0.016 (4)	0.005 (4)	−0.009 (5)
C5	0.059 (5)	0.048 (5)	0.045 (4)	0.001 (4)	−0.010 (4)	0.003 (4)
C7	0.057 (5)	0.056 (5)	0.056 (5)	0.025 (4)	−0.007 (4)	−0.009 (4)
C8	0.052 (5)	0.075 (7)	0.061 (6)	0.012 (5)	−0.002 (4)	0.004 (5)
C11	0.109 (5)	0.068 (3)	0.082 (4)	−0.007 (3)	−0.009 (3)	0.008 (3)
C17	0.044 (4)	0.046 (5)	0.053 (5)	0.007 (4)	0.010 (4)	0.007 (4)
C16	0.036 (4)	0.093 (8)	0.048 (5)	0.013 (4)	−0.004 (4)	0.010 (5)
C13	0.039 (4)	0.063 (6)	0.064 (6)	0.007 (4)	0.002 (4)	−0.002 (5)
C14	0.041 (5)	0.098 (8)	0.065 (7)	0.015 (5)	0.018 (5)	−0.004 (6)
C15	0.043 (5)	0.162 (13)	0.053 (6)	0.023 (7)	0.012 (5)	−0.015 (8)
C12	0.038 (4)	0.058 (5)	0.052 (5)	0.002 (4)	−0.003 (4)	0.004 (4)
C18	0.049 (5)	0.070 (7)	0.084 (8)	0.000 (5)	0.023 (5)	−0.013 (6)
C29	0.055 (5)	0.038 (4)	0.054 (5)	−0.007 (4)	0.013 (4)	0.003 (3)
C30	0.116 (11)	0.040 (5)	0.051 (6)	0.003 (6)	0.010 (6)	0.001 (4)
C31	0.094 (9)	0.056 (6)	0.069 (7)	−0.008 (6)	−0.017 (7)	−0.006 (6)
C33	0.063 (6)	0.069 (7)	0.060 (6)	−0.018 (5)	0.016 (5)	0.013 (6)
C32	0.067 (7)	0.045 (6)	0.121 (12)	−0.028 (5)	−0.010 (7)	0.006 (7)
C34	0.048 (4)	0.042 (4)	0.032 (4)	−0.011 (3)	−0.002 (3)	0.004 (3)
C35	0.065 (6)	0.054 (5)	0.033 (4)	−0.006 (4)	−0.006 (4)	−0.006 (4)
N2	0.032 (3)	0.047 (4)	0.042 (4)	0.006 (3)	0.009 (3)	0.001 (3)
C37	0.042 (4)	0.041 (4)	0.058 (5)	0.009 (3)	0.004 (4)	0.000 (4)
C40	0.109 (5)	0.068 (3)	0.082 (4)	−0.007 (3)	−0.009 (3)	0.008 (3)
C42	0.109 (5)	0.068 (3)	0.082 (4)	−0.007 (3)	−0.009 (3)	0.008 (3)
C38	0.109 (5)	0.068 (3)	0.082 (4)	−0.007 (3)	−0.009 (3)	0.008 (3)
C39	0.109 (5)	0.068 (3)	0.082 (4)	−0.007 (3)	−0.009 (3)	0.008 (3)
C41	0.109 (5)	0.068 (3)	0.082 (4)	−0.007 (3)	−0.009 (3)	0.008 (3)
O4	0.046 (3)	0.078 (5)	0.038 (3)	−0.025 (3)	0.016 (3)	−0.022 (3)
C3	0.048 (5)	0.054 (5)	0.054 (5)	−0.002 (4)	−0.008 (4)	0.016 (4)
Cl1	0.0447 (13)	0.0551 (16)	0.0792 (18)	−0.0145 (12)	0.0057 (12)	0.0028 (13)
Cl2	0.0498 (15)	0.075 (2)	0.0761 (19)	0.0215 (15)	−0.0071 (13)	−0.0090 (16)
Hg1	0.04012 (18)	0.04296 (19)	0.04054 (18)	0.00013 (15)	0.00241 (10)	−0.00068 (16)

### Geometric parameters (Å, º)

**Table:**

S1—O2	1.446 (6)	C18—H18	0.9300
S1—O3	1.465 (5)	C29—C34	1.374 (13)
S1—O4	1.473 (7)	C29—C30	1.422 (15)
S1—O1	1.491 (5)	C29—H29	0.9300
O1—Hg1	2.433 (6)	C30—C31	1.37 (2)
N1—C2	1.508 (12)	C30—H30	0.9300
N1—C35	1.523 (12)	C31—C32	1.34 (2)
N1—H1A	0.9000	C31—H31	0.9300
N1—H1B	0.9000	C33—C34	1.386 (13)
C2—C4	1.514 (12)	C33—C32	1.40 (2)
C2—H2A	0.9700	C33—H33	0.9300
C2—H2B	0.9700	C32—H32	0.9300
C4—C6	1.364 (13)	C34—C35	1.492 (12)
C4—C5	1.382 (13)	C35—H35A	0.9700
C6—C18	1.390 (14)	C35—H35B	0.9700
C6—H6	0.9300	N2—C3	1.477 (12)
C5—C7	1.396 (14)	N2—C37	1.539 (11)
C5—H5	0.9300	N2—H2C	0.9000
C7—C8	1.385 (17)	N2—H2D	0.9000
C7—H7	0.9300	C37—H37A	0.9700
C8—C18	1.367 (17)	C37—H37B	0.9700
C8—H8	0.9300	C40—C39	1.32 (2)
C11—C42	1.36 (2)	C40—H40	0.9300
C11—C40	1.39 (3)	C42—C41	1.51 (2)
C11—C3	1.536 (19)	C42—H42	0.9300
C17—C16	1.374 (15)	C38—C41	1.34 (2)
C17—C12	1.375 (13)	C38—C39	1.34 (2)
C17—C37	1.459 (12)	C38—H38	0.9300
C16—C15	1.388 (14)	C39—H39	0.9300
C16—H16	0.9300	C41—H41	0.9300
C13—C14	1.363 (18)	O4—Hg1^i^	2.533 (7)
C13—C12	1.387 (14)	C3—H3A	0.9700
C13—H13	0.9300	C3—H3B	0.9700
C14—C15	1.392 (18)	Cl1—Hg1	2.327 (3)
C14—H14	0.9300	Cl2—Hg1	2.331 (3)
C15—H15	0.9300	Hg1—O4^ii^	2.533 (7)
C12—H12	0.9300		
			
O2—S1—O3	111.6 (4)	C31—C30—C29	120.6 (13)
O2—S1—O4	110.3 (4)	C31—C30—H30	119.7
O3—S1—O4	110.0 (4)	C29—C30—H30	119.7
O2—S1—O1	108.5 (4)	C32—C31—C30	118.3 (12)
O3—S1—O1	108.3 (3)	C32—C31—H31	120.8
O4—S1—O1	107.9 (4)	C30—C31—H31	120.8
S1—O1—Hg1	115.2 (3)	C34—C33—C32	118.5 (11)
C2—N1—C35	114.7 (7)	C34—C33—H33	120.7
C2—N1—H1A	108.6	C32—C33—H33	120.7
C35—N1—H1A	108.6	C31—C32—C33	123.2 (11)
C2—N1—H1B	108.6	C31—C32—H32	118.4
C35—N1—H1B	108.6	C33—C32—H32	118.4
H1A—N1—H1B	107.6	C29—C34—C33	119.5 (9)
N1—C2—C4	114.5 (7)	C29—C34—C35	121.3 (8)
N1—C2—H2A	108.6	C33—C34—C35	119.1 (9)
C4—C2—H2A	108.6	C34—C35—N1	112.8 (7)
N1—C2—H2B	108.6	C34—C35—H35A	109.0
C4—C2—H2B	108.6	N1—C35—H35A	109.0
H2A—C2—H2B	107.6	C34—C35—H35B	109.0
C6—C4—C5	120.5 (9)	N1—C35—H35B	109.0
C6—C4—C2	120.3 (9)	H35A—C35—H35B	107.8
C5—C4—C2	119.0 (8)	C3—N2—C37	111.9 (7)
C4—C6—C18	120.1 (10)	C3—N2—H2C	109.2
C4—C6—H6	120.0	C37—N2—H2C	109.2
C18—C6—H6	120.0	C3—N2—H2D	109.2
C4—C5—C7	118.7 (9)	C37—N2—H2D	109.2
C4—C5—H5	120.7	H2C—N2—H2D	107.9
C7—C5—H5	120.7	C17—C37—N2	111.8 (8)
C8—C7—C5	121.1 (10)	C17—C37—H37A	109.2
C8—C7—H7	119.4	N2—C37—H37A	109.2
C5—C7—H7	119.4	C17—C37—H37B	109.2
C18—C8—C7	118.7 (11)	N2—C37—H37B	109.2
C18—C8—H8	120.7	H37A—C37—H37B	107.9
C7—C8—H8	120.7	C39—C40—C11	121.3 (18)
C42—C11—C40	120.2 (16)	C39—C40—H40	119.4
C42—C11—C3	121.2 (18)	C11—C40—H40	119.4
C40—C11—C3	118.4 (15)	C11—C42—C41	117.8 (17)
C16—C17—C12	117.2 (8)	C11—C42—H42	121.1
C16—C17—C37	122.0 (9)	C41—C42—H42	121.1
C12—C17—C37	120.7 (9)	C41—C38—C39	124.0 (15)
C17—C16—C15	121.1 (9)	C41—C38—H38	118.0
C17—C16—H16	119.5	C39—C38—H38	118.0
C15—C16—H16	119.5	C40—C39—C38	120.3 (19)
C14—C13—C12	118.2 (10)	C40—C39—H39	119.8
C14—C13—H13	120.9	C38—C39—H39	119.8
C12—C13—H13	120.9	C38—C41—C42	115.8 (14)
C13—C14—C15	120.2 (10)	C38—C41—H41	122.1
C13—C14—H14	119.9	C42—C41—H41	122.1
C15—C14—H14	119.9	S1—O4—Hg1^i^	134.0 (4)
C16—C15—C14	119.8 (11)	N2—C3—C11	112.1 (10)
C16—C15—H15	120.1	N2—C3—H3A	109.2
C14—C15—H15	120.1	C11—C3—H3A	109.2
C17—C12—C13	123.4 (10)	N2—C3—H3B	109.2
C17—C12—H12	118.3	C11—C3—H3B	109.2
C13—C12—H12	118.3	H3A—C3—H3B	107.9
C8—C18—C6	120.8 (11)	Cl1—Hg1—Cl2	152.43 (10)
C8—C18—H18	119.6	Cl1—Hg1—O1	94.47 (15)
C6—C18—H18	119.6	Cl2—Hg1—O1	112.27 (16)
C34—C29—C30	119.7 (10)	Cl1—Hg1—O4^ii^	100.19 (18)
C34—C29—H29	120.2	Cl2—Hg1—O4^ii^	90.91 (17)
C30—C29—H29	120.2	O1—Hg1—O4^ii^	80.8 (2)
			
O2—S1—O1—Hg1	15.7 (4)	C30—C29—C34—C35	176.0 (10)
O3—S1—O1—Hg1	−105.6 (4)	C32—C33—C34—C29	2.1 (17)
O4—S1—O1—Hg1	135.3 (4)	C32—C33—C34—C35	−175.3 (11)
C35—N1—C2—C4	65.4 (10)	C29—C34—C35—N1	65.7 (12)
N1—C2—C4—C6	−102.6 (10)	C33—C34—C35—N1	−116.9 (10)
N1—C2—C4—C5	81.8 (10)	C2—N1—C35—C34	68.1 (10)
C5—C4—C6—C18	−0.2 (16)	C16—C17—C37—N2	62.6 (13)
C2—C4—C6—C18	−175.8 (10)	C12—C17—C37—N2	−120.4 (10)
C6—C4—C5—C7	0.2 (14)	C3—N2—C37—C17	59.3 (10)
C2—C4—C5—C7	175.9 (8)	C42—C11—C40—C39	−5 (3)
C4—C5—C7—C8	1.5 (14)	C3—C11—C40—C39	179.8 (14)
C5—C7—C8—C18	−3.3 (16)	C40—C11—C42—C41	2 (2)
C12—C17—C16—C15	0.5 (19)	C3—C11—C42—C41	177.3 (13)
C37—C17—C16—C15	177.5 (13)	C11—C40—C39—C38	8 (2)
C12—C13—C14—C15	4 (2)	C41—C38—C39—C40	−8 (3)
C17—C16—C15—C14	1 (3)	C39—C38—C41—C42	5 (2)
C13—C14—C15—C16	−3 (3)	C11—C42—C41—C38	−2 (2)
C16—C17—C12—C13	0.3 (16)	O2—S1—O4—Hg1^i^	−97.7 (6)
C37—C17—C12—C13	−176.8 (9)	O3—S1—O4—Hg1^i^	25.8 (7)
C14—C13—C12—C17	−2.5 (17)	O1—S1—O4—Hg1^i^	143.8 (5)
C7—C8—C18—C6	3.3 (18)	C37—N2—C3—C11	−170.1 (11)
C4—C6—C18—C8	−1.6 (19)	C42—C11—C3—N2	102.8 (16)
C34—C29—C30—C31	−0.5 (18)	C40—C11—C3—N2	−82.1 (16)
C29—C30—C31—C32	1 (2)	S1—O1—Hg1—Cl1	92.6 (3)
C30—C31—C32—C33	−1 (2)	S1—O1—Hg1—Cl2	−80.6 (3)
C34—C33—C32—C31	−1 (2)	S1—O1—Hg1—O4^ii^	−167.8 (4)
C30—C29—C34—C33	−1.3 (15)		

Symmetry codes: (i) *x*, −*y*+1, *z*+1/2; (ii) *x*, −*y*+1, *z*−1/2.

### Hydrogen-bond geometry (Å, º)

**Table:**

*D*—H···*A*	*D*—H	H···*A*	*D*···*A*	*D*—H···*A*
N1—H1*A*···O1^iii^	0.90	2.44	2.920 (9)	114
N1—H1*A*···O3^iii^	0.90	2.29	3.037 (10)	141
N1—H1*B*···O3^iv^	0.90	1.90	2.766 (9)	161
N2—H2*C*···O4^i^	0.90	2.32	3.043 (10)	137
N2—H2*C*···O1^i^	0.90	2.12	2.857 (9)	139
C37—H37*A*···Cl2^i^	0.97	2.85	3.716 (10)	149

Symmetry codes: (i) *x*, −*y*+1, *z*+1/2; (iii) *x*+1/2, −*y*+1/2, *z*+1/2; (iv) *x*+1/2, *y*−1/2, *z*.
